# Synergistic effect of electric stimulation and mesenchymal stem cells against Parkinson's disease

**DOI:** 10.18632/aging.103477

**Published:** 2020-08-24

**Authors:** Chunhui Yang, Yiqing Qiu, Yuan Qing, Jinyu Xu, Wei Dai, Xiaowu Hu, Xi Wu

**Affiliations:** 1 Department of Neurosurgery, Changhai Hospital, The Second Military Medical University, Shanghai 2000433, China

**Keywords:** Parkinson's disease, electroconvulsive therapy, MSCs, MPTP

## Abstract

Electroconvulsive therapy (ECT) has known beneficial effects on the core motor symptoms of Parkinson's disease (PD), likely through induction of dopamine release and sensitivity of dopamine receptors. Mesenchymal stem cells (MSCs) can salvage loss of dopamine in PD through their differentiation into dopaminergic neurons. However, it is not known if combined ECT and MSC transplantation may have a synergistic effect against PD. Here, we showed that ECT significantly increased the differentiation of the transplanted MSCs into dopaminergic neurons in a 1-methyl-4-phenyl-1,2,3,6-tetrahydropyridine (MPTP)-induced mouse model of PD. On the other hand, transplantation of MSCs significantly increased dopamine levels after ECT. Co-application of ECT and MSC transplantation generated a synergistic effect through increases in dopamine and decreases in pro-inflammatory cytokines, resulting in significantly attenuated defect in stepping test and rotational behavior in MPTP-mice. Together, our data suggest that combined ECT and MSC transplantation can be a valuable treatment of PD.

## INTRODUCTION

Parkinson’s disease (PD) is a chronic and progressive disease that affects aged people, and is characterized with progressive loss of dopaminergic neurons, which leads to debilitating symptoms of bradykinesia, cogwheel rigidity, immobility and resting tremor [1]. Many PD patients eventually develop suboptimal responses to the current popular pharmacological treatments including L-dopa, dopamine agonists and anticholinergic drugs [2–5], likely due to compromised sensitivity of postsynaptic dopaminergic receptors [6–9]. Hence, alternative approaches should be applied in the treatment of PD.

Both electroconvulsive therapy (ECT) [10] and transplantation of mesenchymal stem cells (MSCs) [11] are attractive strategies against PD, since they have obtained satisfactory therapeutic outcomes in both animal studies [12, 13] and clinic [14–18]. MSCs are adult stem cells which have demonstrated therapeutic potential in treating PD animals or patients, owing to their strong differentiating competence, migratory capacity and production of trophic molecules to boost neuronal cell regeneration and inhibit neuronal cell death [11]. The precise mechanisms by which MSCs exert their effects against development of PD are not completely understood. Nevertheless, differentiation of MSCs into tyrosine hydroxylase (TH)-expressing dopaminergic cells is believed to be a reason of the behavioral recovery in parkinsonian mice [19]. The beneficial effects of ECT on PD have also been documented as early as 1947, and most reports to date have confirmed the beneficial effects of ECT on the motor symptoms in PD [20].

Although both ECT and MSC transplantation are demonstrative therapy for PD, their combined effects on PD have not been assessed. Of note, ECT has been shown to affect neuronal progenitor cell differentiation [21], while MSCs are well-known for their effects on inflammatory status initiated by ECT application [22]. Hence, we studied the combined effects of ECT and MSCs on PD in the current study.

## RESULTS

### Preparation of genetically labeled MSCs

**Figure 1:**
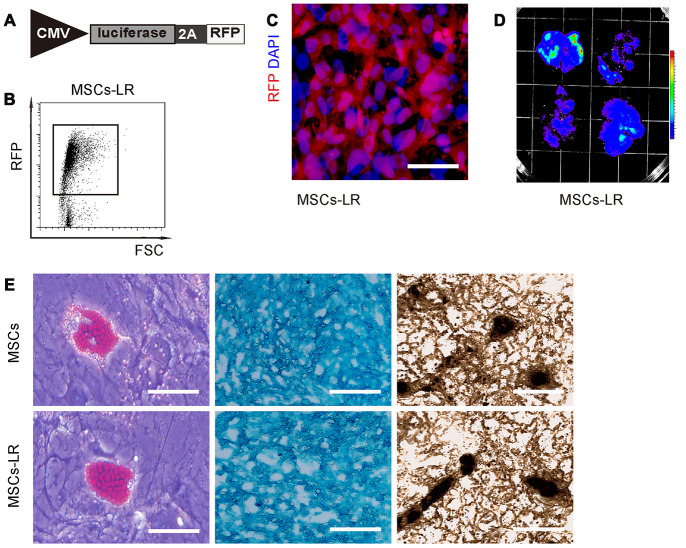
**Preparation of genetically labeled MSCs.** (**A**) Schematic of lentivirus carrying luciferase and RFP reporters with an 2A connector under the control of a CMV promoter. (**B**) Flow cytometry for transduced MSCs (MSC-LR) for RFP. (**C**) MSCs-LR in cell culture, showing RFP label and nuclei by DAPI staining. (**D**) Bioluminescence was detected in MSCs-LR cells after luciferin exposure. (**E**) Osteocyte-, adipocyte- and chondrocyte- differentiation of MSCs-LR and control MSCs, validated by Von Kossa staining, Oil red O staining and Alcian blue staining, respectively. *p<0.05. N=5. Scale bar in panel C is 20μm and in panel D is 100μm.

In order to assess the combined effects of ECT and MSC transplantation on PD, we prepared MSCs that were genetically labeled with luciferase and RFP, to allow in vivo visualization of the transplanted cells and isolation of the transplanted cells from the receipt brain by flow cytometry, respectively. Thus, isolated mouse MSCs were transduced with lentivirus carrying luciferase and RFP reporters with an 2A connector under the control of a CMV promoter (Figure 1A). After transduction, the red fluorescent cells were purified by flow cytometry (Figure 1B), and then put in culture. The prepared cells were red fluorescent (Figure 1C), and the bioluminescence can be detected after luciferin exposure (Figure 1D). These data confirmed that these MSCs successfully carried both luciferase and RFP reporters (MSCs-LR, which was used in this study and simplified as MSCs from now on). To confirm that the genetically labeled MSCs did not lose differentiation potential, they were subjected to differentiation assay, which confirmed their maintenance of differentiation potential into osteocyte, adipocyte and chondrocyte, by Von Kossa staining, Oil red O staining and Alcian blue staining, respectively (Figure 1E). Thus, these cells were readily used in our study.

### Experimental schematic

**Figure 2:**
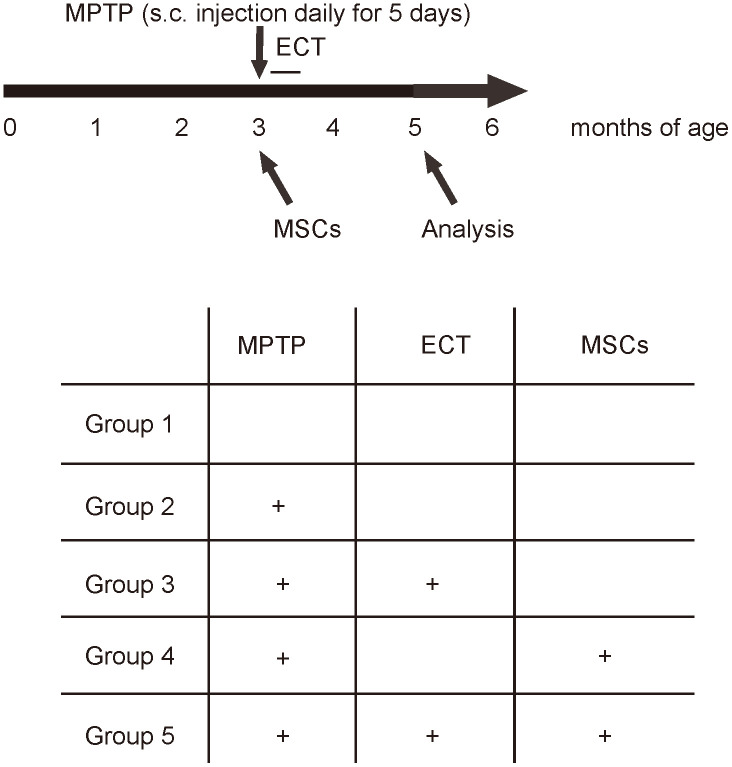
**Experimental Schematic. The CBA mice were randomly assigned into 5 groups.** Group1 (n=10): to receive daily subcutaneous injection of saline for 5 days and one intracranial injection of saline (saline); Group 2 (n = 10): to receive daily subcutaneous injection of 20mg/kg MPTP (Sigma-Aldrich, St. Louis, MO, USA) for 5 days and one intracranial injection of saline (MPTP); Group 3 (n = 10): to receive daily subcutaneous injection of 20mg/kg MPTP for 5 days, one intracranial injection of saline and daily ECT treatment (an electrical current of 80 mC (80 mA, 50 Hz, 1s duration and 0.5ms pulse width) through ear clip electrodes) for 8 days (MPTP+ECT); Group 4 (n = 10): to receive daily subcutaneous injection of 20mg/kg MPTP for 5 days and one intracranial injection of 10^12^ MSCs from isogeneic mice (MPTP+MSCs); Group 5 (n = 10): to receive daily subcutaneous injection of 20mg/kg MPTP for 5 days, one intracranial injection of 10^12^ MSCs from isogeneic mice and daily ECT treatment (an electrical current of 80 mC (80 mA, 50 Hz, 1s duration and 0.5ms pulse width) through ear clip electrodes) for 8 days (MPTP+ECT+MSCs). All the mice were kept for another 2 months, and then subjected to rational behavior test, stepping test for assessing behavioral disorder. Afterwards, the mice were sampled for analyzing histology and signaling to dissect underlying mechanisms.

MPTP is converted to 1-methyl-4-phenylpyridinium by monoamine oxidase-B in astrocytes and then transported into dopaminergic neurons by dopamine transporter to create neurotoxicity. We used MPTP to generate PD model in mice. The CBA mice were randomly assigned into 5 groups: Group1: saline; Group 2: MPTP; Group 3: MPTP+ECT; Group 4: MPTP+MSCs; Group 5: MPTP+ECT+MSCs. All the mice were kept for another 2 months, and then subjected to rational behavior test, stepping test for assessing behavioral disorder. Afterwards, the mice were sampled for analyzing histology and signaling to dissect underlying mechanisms (Figure 2).

### Combined ECT and MSCs synergistically alleviate PD-associated behavior disorder

**Figure 3:**
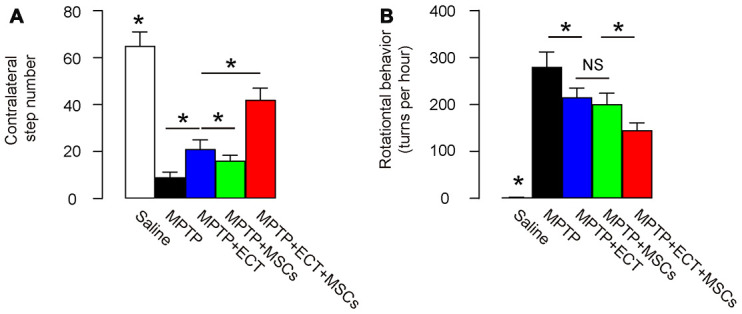
**Combined ECT and MSCs synergistically alleviate PD-associated behavior disorder.** (**A**, **B**) Two months after treatment, behavioral disorder in MPTP-mice was assessed with two tests, stepping test (**A**) and rotational behavioral test (**B**). *p<0.05. NS: non-significant. N=10.

Two months after treatment, we assessed the behavioral disorder in MPTP-mice with two tests, stepping test and rotational behavioral test. In the stepping test, we found that MPTP significantly decreased the contralateral step number, which was attenuated by either ECT or MSC transplantation (Figure 3A). The effects of ECT appeared to be more pronounced than the effects of MSCs (Figure 3A). However, the combined treatment of ECT and MSCs exerted significantly stronger effects than either ECT alone or MSCs alone (Figure 3A). Similar results were obtained from rotational behavioral test, in which MPTP significantly increased the rotational turns in mice, which were attenuated by either ECT or MSC transplantation (Figure 3B). Here, no difference was detected between the effects of ECT and the effects of MSCs on the turn number (Figure 3B). However, the combined treatment of ECT and MSCs exerted significantly stronger effects than either ECT alone or MSCs alone (Figure 3B). Together, these data suggest that while ECT or MSCs alone significantly alleviates the PD-associated behavior disorder, the combined ECT and MSCs have a synergistic effect.

### ECT increases proliferation and survival of grafted MSCs

**Figure 4:**
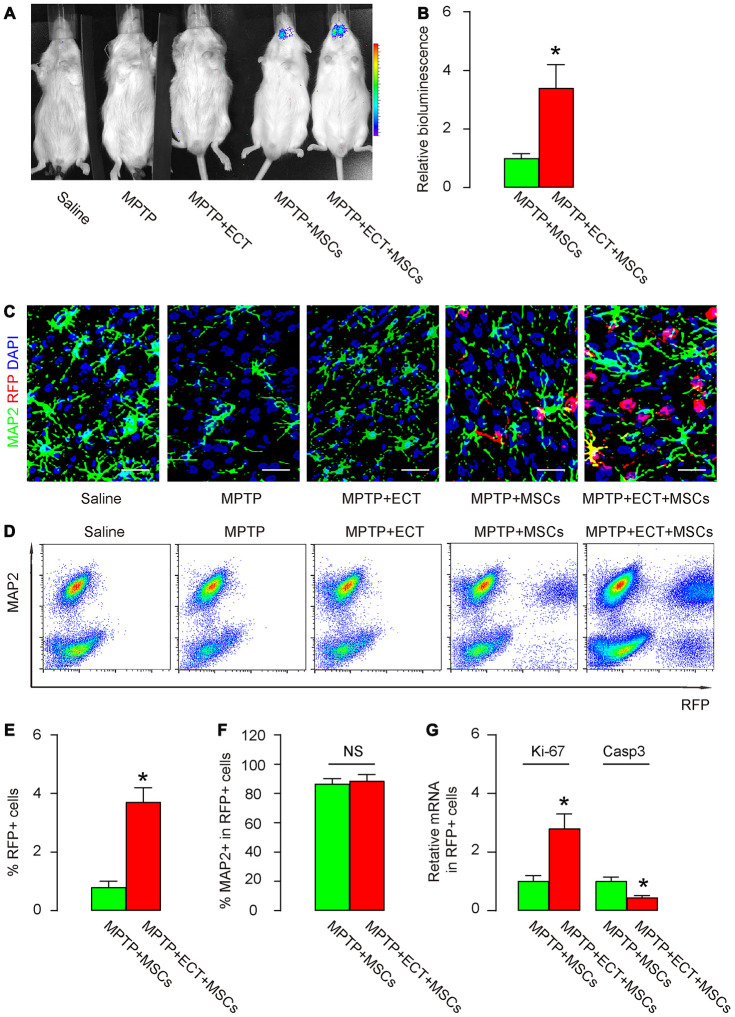
**ECT increases proliferation and survival of grafted MSCs.** (**A**, **B**) Bioluminescence test for detecting grafted MSCs in mice two months after treatment, shown by representative images (**A**) and by quantification (**B**). (**C**) Representative immunohistochemical images for MAP2 and RFP in mouse brain two months after treatment. (**D**) Representative flow charts for MAP2 and RFP for mouse brain cells two months after treatment. (**E**) Quantification of percentage of RFP+ cells in total cells. (**F**) Quantification of percentage of MAP2+ cells in total RFP+ cells. (**G**) RT-qPCR for Ki-67 and Casp3 in total RFP+ cells. *p<0.05. NS: non-significant. N=10. Scale bars are 20μm.

Next, we analyzed the number and phenotypic alteration in grafted MSCs. First, bioluminescence test was performed, showing exclusive luciferase signals in group MPTP+MSCs and group MPTP+ECT+MSCs (Figure 4A). Interestingly, the luciferase signals in group MPTP+ECT+MSCs were significantly higher than in group MPTP+MSCs (Figure 4A, 4B). These data suggest that ECT may increase the number of the granted MSCs. To prove it and further examine the differentiation of grafted MSCs into neuronal cells, we did 2 experiments. First, we stained the pars compacta of substantia nigra region of the mouse brain for MAP2, a mature neuronal cell marker and RFP (unique for grafted MSCs). While no RFP cells were detected in group saline, MPTP or MPTP+ECT, RFP+ cells were detected in MPTP+MSCs and group MPTP+ECT+MSCs (Figure 4C), and group MPTP+ECT+MSCs appeared to have more RFP+ cells than group MPTP+MSCs (Figure 4C). In the second approach, we dissociated the pars compacta of substantia nigra region of mouse brain tissue and analyzed for MAP2 and RFP by flow cytometry (Figure 4D). Again, while no RFP cells were detected in group saline, MPTP or MPTP+ECT, RFP+ cells were detected in MPTP+MSCs and group MPTP+ECT+MSCs (Figure 4D). Group MPTP+ECT+MSCs contained significantly more RFP+ cells than group MPTP+MSCs (Figure 4D, 4E). The ratio of MAP2+ cells in the total RFP+ cells was, however, not different between group MPTP+MSCs and group MPTP+ECT+MSCs (Figure 4F), suggesting that ECT may increase the grafted MSC number rather than their differentiation. Finally, we examined a proliferation gene Ki-67 and an apoptosis gene Caspase3 in the purified RFP+ cell population. We detected significantly increases in Ki-67 and significantly decreases in Caspase3 in group MPTP+ECT+MSCs than in group MPTP+MSCs (Figure 4G). These data suggest that ECT increases proliferation and survival of grafted MSCs.

### Combined ECT and MSCs synergistically increase dopamine levels

**Figure 5:**
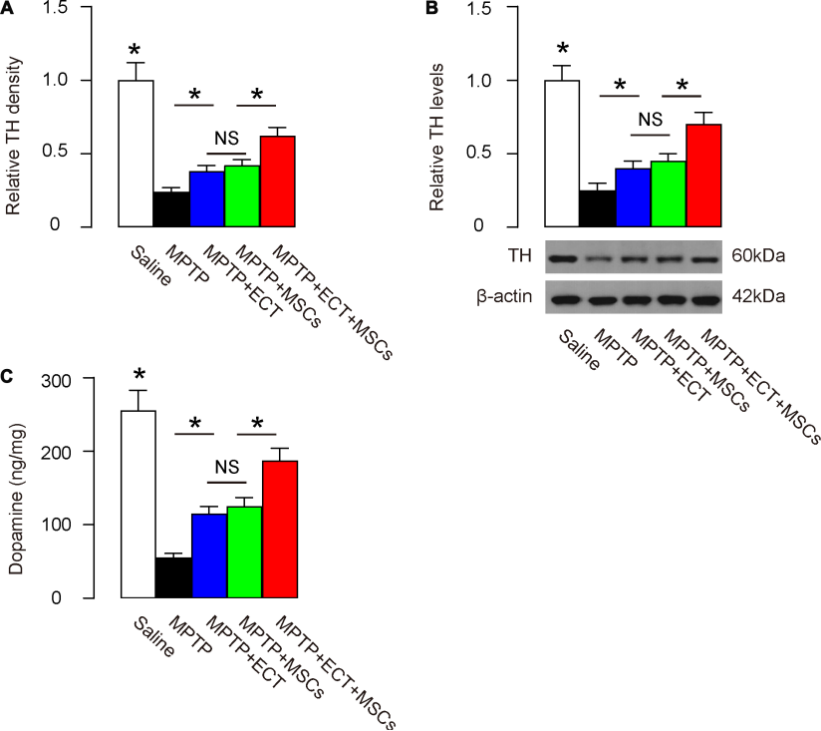
**Combined ECT and MSCs synergistically increase dopamine levels.** (**A**) Quantification of TH density by immunohistochemistry. (**B**) Western blotting for TH in brain tissue. (**C**) ELISA for dopamine levels in brain tissue. *p<0.05. NS: non-significant. N=10. Scale bars are 20μm.

Parkinson’s disease is characterized with progressive degeneration of dopaminergic neurons, where tyrosine hydroxylase (TH) catalyzes the formation of L-dihydroxyphenylalanine (L-DOPA) as the rate-limiting step in the biosynthesis of DA [23, 24]. Thus, we examined the levels of TH in mouse brain at 2 months after treatment. By immunohistochemistry, we found that the compromised TH density was attenuated by either ECT or MSC transplantation (Figure 5A). Here, no difference was detected between the effects of ECT and the effects of MSCs on TH (Figure 5A). However, the combined treatment of ECT and MSCs exerted significantly stronger effects on the salvage of TH than either ECT alone or MSCs alone (Figure 5A). Similar results were obtained from quantitative analysis of TH protein levels in the mouse brain. MPTP significantly decreased the TH levels, which was attenuated by either ECT or MSC transplantation (Figure 5B). Here, no difference was detected between the effects of ECT and the effects of MSCs on TH protein (Figure 5B). However, the combined treatment of ECT and MSCs exerted significantly stronger salvaging effects on TH protein than either ECT alone or MSCs alone (Figure 5B). The dopamine levels were also measured, showing consistent results with TH data (Figure 5C). Together, these data suggest that while ECT or MSCs alone significantly alleviates the PD-associated loss of TH and dopamine, the combined ECT and MSCs have a synergistic effect.

### MSCs reduces pro-inflammatory cytokines

**Figure 6:**
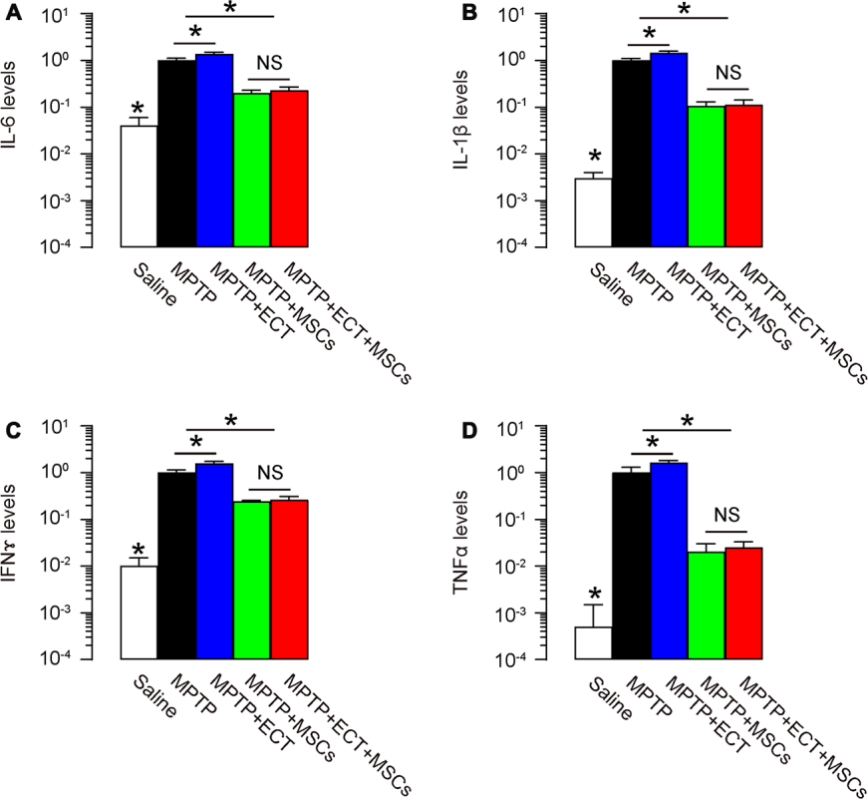
**MSCs reduces pro-inflammatory cytokines.** (**A**–**D**) ELISA for pro-inflammatory cytokine IL-6 (**A**), IL-1β (**B**), IFNɤ (**C**) and TNFα (**D**) in mouse brain. *p<0.05. NS: non-significant. N=10.

Pro-inflammatory cytokines play critical roles in the PD development and ECT-associated side effects. Hence, we analyzed expression of some pro-inflammatory cytokines in the mouse brain. We detected high levels for pro-inflammatory cytokine IL-6 (Figure 6A), IL-1β (Figure 6B), IFNɤ (Figure 6C) and TNFα (Figure 6D) in MPTP-treated mouse brain, while MSCs, but not ECT, significantly attenuated production of these cytokines. Interestingly, the slight increases in pro-inflammatory cytokines by ECT (group MPTP+ECT versus group MPTP, p<0.05) were abolished by MSCs (group MPTP+ECT+MSCs versus group MPTP+MSCs, no significance) (Figure 6A–6D). Thus, MSCs reduces pro-inflammatory cytokines induced by MPTP and ECT.

## DISCUSSION

Both electroconvulsive therapy (ECT) [10] and transplantation of MSCs [11] have demonstrative effects against PD [14–18]. Surprisingly, their combined effects on PD have not been assessed, given that ECT is capable of promoting neuronal progenitor cell differentiation [21], while MSCs have an antagonizing effect against harmful inflammation [22].

Indeed, in the current study, we found out that ECT and MSCs appeared to synergistically inhibit the progression and severity of PD-features in mice. The combined ECT and MSCs synergistically alleviated PD-associated behavior disorder. This outcome should result from a combined effect of ECT and MSCs on the alleviation of the corresponding damages, based on our analysis on the MAP2+ cells, TH density and dopamine levels in brains. First, ECT appeared to enhance the survival and proliferation of the grafted MSCs, resulting in increases in the number of MSCs after transplantation. Ki-67 is a proliferation marker that plays a substantial role in an active cell cycle, while Casp3 is a key factor in the apoptosis pathway. Increases in Ki-67 levels and decreases in Casp3 levels in MSCs suggest that the increases in total MSC number may be due to both the increases in MSC proliferation and the decreases in cellular death. Of note, the differentiation potential of MSCs, which was determined by the ratio of MAP2+ versus MAP- cells in the total RFP+ MSCs, appeared not to be affected by ECT. However, the increases in the grafted MSCs with proliferative potential could increase the total number of differentiated dopaminergic neuronal cells, which subsequently exert a robust protective effect against PD.

On the other hand, MSCs appeared to benefit ECT-mediated correction of motor symptoms in PD, likely through suppression of inflammation [11]. It is well-known that besides antagonizing PD-associated motor disorder [20], ECT also created a tissue damage niche to recruit inflammatory cells that mediate immune response [22]. MSCs, which are potent inhibitors for inflammation, could suppress the generation of inflammatory cytokines caused by MPTP/PD and by ECT. For example, it was known that MSCs could be activated by TNFα and then produced and secreted anti-inflammatory cytokines IL-10 and transforming growth factor β [25]. Therefore, ECT and MSCs appeared to synergistically function in the model of PD, reinforced each other’s potential, and eventually caused a significantly better outcome of the treatment in mice.

The MPTP model is so far the most widely used animal model for human PD [26]. MPTP is a lipophilic protoxin capable of crossing the blood-brain barrier. In brain, MPTP is taken up by the astrocytes to be catalyzed by monoamine oxidase-B to generate 1-methyl-4-phenylpyridinium, which is then taken via dopamine transporter into dopaminergic neurons [26]. This model faithfully mimicked many features of the biological and pathological changes in PD in humans. In future, more models could be tested for verifying the synergistic effects of ECT and MSCs on PD to exclude the possible model-dependence of the current finding.

To the best of our knowledge, it is the first study to show a synergistic effect of ECT and MSCs on PD in rodent models. This strategy may be translated to clinic application after further validations.

## MATERIALS AND METHODS

### Protocol approval

All the experimental procedures in the current study have been approved by the research committee at the Second Military Medical University. All the experiments have been carried out in accordance with the guidelines from the research committee at the Second Military Medical University.

### Mouse treatment

Male CBA mice were obtained from the SLAC Laboratory Animal Co. Ltd (Shanghai, China) and fed on standard pellet chow and water. The littermate mice were used for experiments at age of 3 months-old when they were randomly assigned into 5 groups: Group1 (n=10): to receive daily subcutaneous injection of saline for 5 days and one intracranial injection of saline (saline); Group 2 (n = 10): to receive daily subcutaneous injection of 20mg/kg MPTP (Sigma-Aldrich, St. Louis, MO, USA) for 5 days and one intracranial injection of saline (MPTP); Group 3 (n = 10): to receive daily subcutaneous injection of 20mg/kg MPTP for 5 days, one intracranial injection of saline and daily ECT treatment (an electrical current of 80 mC (80 mA, 50 Hz, 1s duration and 0.5ms pulse width) through ear clip electrodes) for 8 days (MPTP+ECT); Group 4 (n = 10): to receive daily subcutaneous injection of 20mg/kg MPTP for 5 days and one intracranial injection of 10^12^ MSCs from isogeneic mice (MPTP+MSCs); Group 5 (n = 10): to receive daily subcutaneous injection of 20mg/kg MPTP for 5 days, one intracranial injection of 10^12^ MSCs from isogeneic mice and daily ECT treatment (an electrical current of 80 mC (80 mA, 50 Hz, 1s duration and 0.5ms pulse width) through ear clip electrodes) for 8 days (MPTP+ECT+MSCs). All the mice were kept for another 2 months, and then subjected to rational behavior test, stepping test and sampling for analyzing histology and cell and molecular biology. Bioluminescence detection system (IVIS imaging system, Xenogen Corp., Alameda, CA, USA; one-minute exposure, binning 8, f-stop 1, FOV 15 cm) was used to detect grafted MSCs in the mouse brain. At analysis, the quantification was done at 10 minutes after intraperitoneal injection of luciferin at 150 mg/kg body weight.

### Isolation and genetic labeling of mouse MSCs

Mouse-derived MSCs were obtained from male CBA mice at 12 weeks of age (SLAC Laboratory Animal Co. Ltd) from the flush-out from the bone cavity. After centrifugation, cells were cultured in Dulbecco’s modified Eagle medium (DMEM, Gibco; Life Technologies, Carlsbad, CA, USA) with 10% fetal bovine serum (FBS, Sigma-Aldrich), 100 U/ml penicillin (Gibco), 100g/ml streptomycin (Gibco), 5 ng/ml bFGF (Gibco) and 5 ng/ml EGF (Gibco). The non-adherent cells were continuously discarded for 6 passages to obtain the adherent MSCs, the phenotype of which were confirmed by featured expression of surface markers in flow cytometry (positive for CD73, CD90 and CD105, negative for CD34, CD45 and HLA-DR). Differentiation capacity of MSCs was assessed by inducing osteocyte, adipocyte and chondrocyte differentiation with corresponding kits (American Type Culture Collection (ATCC), Rockville, MD, USA; Catalog number: PCS-500-052, PCS-500-050 and PCS-500-051), and evaluated by Von Kossa staining, Oil red O staining and Alcian blue staining, respectively. The MSCs were genetically labeled by lentiviruses that carry luciferase and red fluorescent protein (RFP) reporters with an 2A connector under the control of a CMV promoter. A pcDNA3.1-CMV-luciferase-2A-RFP plasmid was used as a backbone for preparation of lentivirus (Clontech, Mountain View, CA, USA). Transfection was conducted with Lipofectamine 3000 (Invitrogen, Carlsbad, USA) in seeded HEK293T cells (ATCC) with 5 μg of pcDNA3.1-CMV-luciferase-2A-RFP and each of packaging plasmids (REV, pMDL and VSV-G). The supernatant was removed 48 hours after transfection and filtered through the 0.45 μm syringe filter, after which the lentivirus in supernatant was further processed, isolated and titrated.

### Flow cytometry

The dissociated brain tissue cells were fixed before analyzing for RFP and MAP2. RFP was detected by endogenous fluorescence. MAP2 was detected with a primary rabbit anti-MAP2 antibody (ab32454, Abcam, San Jose, CA, USA) followed by APC-conjugated anti-rabbit secondary antibody (Jackson ImmunoResearch Labs, West Grove, PA, USA). Flow cytometry data were analyzed and presented with FlowJo software (Flowjo LLC, Ashland, OR, USA).

### Rational behavior test

Apomorphine (Sigma-Aldrich) was prepared at a concentration of 0.5 mg/ml in sterile 0.02% ascorbic acid saline solution in dark. Briefly, the rats were placed in rotometer bowls and secured to count rotation. Once they were acclimated for 15 min, apomorphine (0.5 mg/kg) was given subcutaneously at the back of neck. After 12 min, the number of net rotations (360° contralateral turns) was continuously recorded for 1 hour.

### Stepping test

For assessment of the motor performance of each forepaw, individual mouse was gently situated into the both hands of the experimenter to allow only one forepaw to hung out of the grip. The mice were guided to move in the sideway over a wooden bar (100cm length, over 15 s). The stepping ratio was calculated by dividing the median stepping number of the ipsilateral paw by the median stepping number of the contralateral paw separately for every condition. A mean stepping ratio was calculated.

### Immunohistochemistry, Western blot and ELISA

Animals were perfused with 4% paraformaldehyde (PFA) after the experiments were completed. Brain samples of the pars compacta of substantia nigra region were collected and post-fixed in 4% PFA at 4°C overnight, transferred to 30% sucrose in phosphate-buffered saline (PBS) overnight. The brain tissues were then sectioned on a cryostat in 6μm sections. The sections were stained with fluorescent method. The primary antibodies were rabbit-anti-mouse MAP2 (ab32454, Abcam) and mouse monoclonal anti-mouse TH (SAB4200699, Sigma-Aldrich). RFP was detected by direct fluorescence. DAPI was used to stain nuclei. Secondary antibody is cy2- or cy3-conjugated anti-rabbit or anti-mouse (Jackson ImmunoResearch Labs, West Grove, PA, USA). Total Protein was extracted using RIPA buffer (Sigma-Aldrich). Western blot and ELISA were done routinely. Rabbit-anti-mouse β-actin (ab8227, Abcam) was used as a protein loading control. The protein levels were first normalized to β-actin, and then normalized to the experimental controls. ELISA for mouse dopamine, IL-6, IL-1β, IFNɤ and TNFα was performed with corresponding ELISA kits (R&D Systems, Los Angeles, CA, USA), according to the manufacturer’s instruction.

### RT-qPCR

Total RNA was extracted from clinical specimens or from cultured cells using a miRNeasy mini kit (Qiagen, Hilden, Germany). Complementary DNA (cDNA) was randomly primed from total RNA using the Omniscript reverse transcription kit (Qiagen). Quantitative PCR (RT-qPCR) were performed in duplicates with QuantiTect SYBR Green PCR Kit (Qiagen). Ki-67, cleaved Caspase 3 (Casp3) and β-actin primers were all purchased from Qiagen. Data were collected and analyzed using ^2-ΔΔ^Ct method for quantification of the relative mRNA expression levels. Values of genes were first normalized against β-actin, and then compared to experimental controls.

### Statistical analysis

All statistical analyses were carried out using the SPSS 19.0 statistical software package. All values are depicted as mean ± SD and are considered significant if p < 0.05. All data were statistically analyzed using one-way ANOVA with a Bonferroni correction, followed by Fisher’s Exact Test for comparison of two groups. The number of the animals per group was ten, decided by power test.
